# The cost of prospecting for dispersal opportunities in a social bird

**DOI:** 10.1098/rsbl.2016.0316

**Published:** 2016-06

**Authors:** Sjouke A. Kingma, Jan Komdeur, Martijn Hammers, David S. Richardson

**Affiliations:** 1 School of Biological Sciences, University of East Anglia, Norwich Research Park, Norwich NR4 7TJ, UK; 2 Behavioural and Physiological Ecology, GELIFES, University of Groningen, PO Box 11103, 9700CC Groningen, The Netherlands; 3 Nature Seychelles, PO Box 1310, Mahé, Seychelles

**Keywords:** cooperative breeding, benefits of philopatry, delayed dispersal

## Abstract

Understanding why individuals delay dispersal and become subordinates within a group is central to studying the evolution of sociality. Hypotheses predict that dispersal decisions are influenced by costs of extra-territorial prospecting that are often required to find a breeding vacancy. Little is known about such costs, partly because it is complicated to demonstrate them empirically. For example, prospecting individuals may be of inferior quality already before prospecting and/or have been evicted. Moreover, costs of prospecting are mainly studied in species where prospectors suffer from predation risk, so how costly prospecting is when predators are absent remains unclear. Here, we determine a cost of prospecting for subordinate Seychelles warblers, *Acrocephalus sechellensis*, in a population where predators are absent and individuals return to their resident territory after prospecting. Prospecting individuals had 5.2% lower body mass than non-prospecting individuals. Our evidence suggests this may be owing to frequent attacks by resident conspecifics, likely leading to reduced food intake by prospectors. These results support the hypothesis that energetic costs associated with dispersal opportunities are one factor influencing dispersal decisions and shaping the evolution of delayed dispersal in social animals.

## 1. Introduction

Across a range of vertebrates, sexually mature individuals forego independent breeding while remaining in a group [1–7]. Understanding why such ‘subordinates’ delay dispersal to an independent breeding position is crucial for understanding the evolution of sociality and cooperative breeding [1,2]. Two hypotheses aim to explain delayed dispersal. The ‘ecological-constraints’ hypothesis predicts that delayed dispersal is driven by limited availability of suitable breeding vacancies and the costs associated with finding those [1]. The ‘benefits-of-philopatry’ hypothesis predicts that subordinates remain in a group when the benefits gained in their home territory (e.g. access to resources, protection against predators) exceed those of dispersal [2]. Although both hypotheses highlight the importance of the relative costs of dispersal, empirical tests of such costs are surprisingly rare [3–8].

In many species, individuals aiming to disperse perform extra-territorial prospecting (using their home territory as base) to obtain information about breeding opportunities [5–9]. The associated costs (e.g. physiological costs linked to reduced food intake resulting from vigilance against predators or intra-specific chases) may be important to whether individuals decide to prospect and, consequently, ultimately disperse. However, knowledge about prospecting costs is limited, as assessing condition of prospectors is challenging and may be confounded if prospectors were evicted from their resident territory and/or are of inferior quality in the first place [5]. Similarly, if non-prospecting subordinates engage in energetically costly helping behaviour, direct comparison of their and prospectors' body condition is misleading. Moreover, studies that described costs of prospecting were all conducted in species where adults can be preyed upon. In such species, prospecting may, in addition to being energetically costly, lead to enhanced predation risk and associated stress [3–7]. Thus, it is currently unclear how costly prospecting is when predation is absent. Additionally, individuals may accept to pay higher costs if prospecting yields additional benefits like access to food, refuge from predators or extra-group copulations [7,9]. Thus, because multiple benefits may drive prospecting, not considering these benefits simultaneously may lead to underestimation of the importance of the costs of prospecting in explaining delayed dispersal. These complications require consideration when determining the costs of prospecting.

We assess the energetic costs of prospecting in facultative cooperatively breeding Seychelles warblers, where subordinates of both sexes postpone independent breeding and may engage in prospecting trips from their resident, often natal, territory [10,11]. Prospecting does not yield access to food, refuge or extra-group mating, but improves an individual's chances of breeding independently (73% of prospectors obtained a breeding position before the next season versus 50% of non-prospecting individuals [12]). Nonetheless, prospecting is rare (approx. 14% of subordinate individuals each season; JK, DSR, SAK 1985--2016, personal observations) and, given the expected increased fitness for subordinates of finding a breeder vacancy, vacancies remain unoccupied surprisingly long in this saturated population (up to 20 days; median 3.5 days [13]). This may suggest that costs inhibit prospecting. Here, we estimate a cost of prospecting by comparing the condition of subordinates caught within their resident territory and during temporary prospecting trips. This can be investigated while ruling out the aforementioned confounding variables (predation, eviction, helping): adult predation is absent at all times (JK, DSR 1985--2016, personal observations), prospectors normally return home and not all subordinates help [9–11]. We also make inferences about the mechanism behind potential costs by assessing whether territory-owners attack intruding prospectors.

## 2. Material and methods

We studied the population of approximately 320 Seychelles warblers living in 110–120 territories on Cousin Island, Seychelles (29 ha; 04°20′S, 55°40′E) from June–September 2003 to 2014. Approximately 50% of pairs live with one to four subordinates that may help incubating and feeding nestlings [11]. Territories are defended against intruding conspecifics year-round, and boundaries are determined based on the birds' activity and border disputes. Breeder vacancies are limited because Seychelles warblers are long-lived (84% annual survival) and the island has been at carrying capacity since 1982 [10,11]. During each season each territory was checked weekly. We determined each bird's status (dominant individuals—based on behavioural interactions including mate-guarding, courtship feeding and other affiliative behaviours [13]—or subordinate), territory, sex (using molecular techniques) and helping behaviour (using 1 h nest-watches during nestling feeding) [10,11,13]. Birds were captured using mistnets (placed in dense vegetation) during regular catching sessions throughout the season (electronic supplementary material), ringed with a unique set of a metal and three-colour rings, and body mass (±0.1 g) and tarsus length (±0.1 mm) were measured [10,11,13]. During these sessions, we opportunistically caught 27 prospecting individuals. We defined prospectors as individuals captured three or more territories from their resident territory; this avoided including individuals from adjacent territories attracted to nearby song playback sometimes used to capture birds (electronic supplementary material).

We determined whether body mass was predicted by whether the individual was caught while prospecting or in their resident territory using a linear mixed model with ‘year’, ‘resident-territory’ and ‘individual’ identity as random variables (to account for consistent temporal, spatial or individual effects) using *lme4* (v. 1.1-8) in R (v. 3.2.0) [14,15]. Tarsus length, sex, age (below 1 year, 1–2 years), time (morning (06.34–10.00), midday (10.00–14.00), afternoon (14.00–19.10)) and month (June/July/August/September) were included as additional explanatory variables. We only included 5–24 months old individuals because younger (just independent; 3–5 months) and older birds rarely prospected (1 of 209 and 0 of 49 catches, respectively). Additionally, we only included non-helpers and helpers caught before the nestling period, because nestling provisioning may be energetically costly and reduce body condition. Of 214 individuals, multiple catches were included for 35 individuals, but results were similar when including only first catches (electronic supplementary material, table S1). Food (arthropods) abundance varies within seasons, but prospectors and resident birds were caught in similar proportions across months (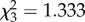, *p* = 0.721).

Territory owners physically attack intruding individuals, and birds caught together with another bird were often involved in intra-specific chases (JK, DSR, SAK 1985–2016, personal observations). We assessed whether the likelihood that individuals were caught with a resident individual was different between prospectors and resident individuals using a generalized linear mixed model. Only ‘individual identity’ was included as random variable, as the model including ‘year’ did not converge. Repeating the analysis with each individual's first catch and ‘year’ as random variable yielded a similar non-significant effect (*β* = 1.232 ± 0.745, *p* = 0.098; see electronic supplementary material).

## 3. Results

**Figure 1.**
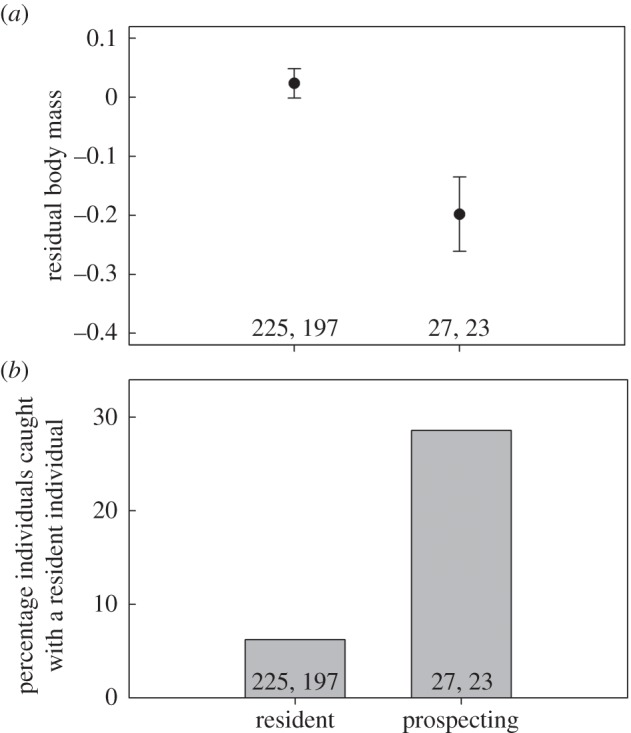
(*a*) Average (±s.e.) body mass of Seychelles warblers caught in their resident territory and while prospecting. Residuals were obtained from a model including tarsus length, sex, and time and month of catching. (*b*) The percentage of catches in which resident and prospecting subordinates were caught with a resident bird. Numbers denote number of catches and individuals, respectively.

We caught 214 different individuals (35 more than once), of which 23 (11%) were caught while prospecting. Prospectors always returned to their resident territory, apart from one individual who was never seen again (see the electronic supplementary material for details). Individuals caught during prospecting had significantly lower body mass than individuals caught in their resident territory, while statistically controlling for other predictors of body mass (table 1 and figure 1*a*). Prospectors were more often caught with a resident individual than non-prospecting subordinates (*β* = 1.460 ± 0.539, *z* = 2.709, *p* = 0.007; figure 1*b*).

**Table 1.** The effect of prospecting on 214 subordinate Seychelles warblers' body mass (including 252 catches).

	*β*	s.e.	*t*	*p*-value
intercept	6.012	1.725	3.484	<0.001
prospecting at time of catch^a^	−0.472	0.140	−3.377	<0.001
sex^b^	0.799	0.143	5.569	<0.001
tarsus length	0.322	0.071	4.532	<0.001
time^c^				
midday	0.258	0.108	2.386	0.018
afternoon	0.540	0.112	4.820	<0.001
month^d^				
July	0.573	0.124	4.620	<0.001
August	0.999	0.134	7.461	<0.001
September	0.907	0.143	6.337	<0.001

Effects relative to ^a^resident, ^b^female, ^c^morning and ^d^June.

## 4. Discussion

Subordinate Seychelles warblers are constrained in independent breeding owing to habitat saturation, and therefore, often remain within another pair's territory. Subordinates can inherit a territory or fill a neighbouring vacancy [10,11], but can improve the likelihood of finding a breeding opportunity by prospecting further afield to obtain information about availability of vacancies (see §1). Our results suggest that prospecting is costly, as it leads to a significantly lowered body condition (figure 1*a*). This result was not the effect of temporal variation in food availability, or prospecting individuals being evicted (prospectors returned home) or being in poorer condition prior to prospecting (within-individual analyses revealed a similar body-mass reduction to the cross-sectional analysis; electronic supplementary material, figure S1). Instead, as in other species [5], the effect likely results from the prospectors having reduced foraging time owing to increased antagonistic interactions with conspecifics (figure 1*b*), while resident subordinates have undisturbed access to resources because of acceptance by breeders (e.g. nepotism [4]). Although the exact duration and frequency of prospecting are unknown and the short-term body-mass reduction does not affect survival [12]*,* it may cause somatic damage or a trade-off with other important life-history decisions like helping behaviour [9]. Regardless of long-term physiological and survival costs, however, individuals require sufficient condition to prospect if prospecting is condition dependent [16]. This may partly explain why prospecting is generally rare [10], and why in some species individuals reduce other costly behaviour (e.g. helping) or gain weight prior to dispersal [17].

Given the absence of evidence for long-term costs of prospecting, it is possible that short-term mass loss is an adaptation to improve manoeuvrability to avoid conspecifics while prospecting. Although we cannot exclude this possibility in our study, in other species where a reduction would be even more beneficial (owing to the presence of predators) body mass loss positively correlates with prospecting duration [5], suggesting a cost rather than an adaptation. Moreover, in several species higher body mass predicts whether individuals prospect [17]. Therefore, although a body-mass reduction associated with prospecting suggests that the behaviour is costly, future studies are needed to explore the possibility of strategic body-mass reduction.

Determining the relative costs of dispersal and benefits of philopatry is crucial to understanding the evolution of sociality [1,2,8]. In Seychelles warblers, reproductive benefits of philopatry are often limited and probably insufficient to induce philopatry (at least in isolation): a substantial number of non-helping philopatric individuals gain no indirect benefits, territory inheritance is rare, and young subordinates rarely obtain parentage [10,11,13]. Moreover, adult predation is absent, so predation risk during prospecting cannot explain delayed dispersal. Finally, prospectors do not gain access to extra-group parentage [13], refuge or food (prospectors are attacked by conspecifics), so the detrimental effect of prospecting can probably be solely attributed to attempted dispersal. Our results suggest that the costs of extra-territory movement are one reason for subordinate Seychelles warblers to delay dispersal beyond sexual maturity, in order to wait until their condition is sufficient to perform prospecting trips or until reproductive opportunities arise. Thus, supporting the ecological-constraints and benefits-of-philopatry hypotheses [1,2], our results suggest that prospecting for dispersal opportunities is energetically costly, which may affect delayed dispersal in this and other species.

## Supplementary Material

Supplementary methods and results

## Data accessibility

Data are deposited in Dryad: http://dx.doi.org/10.5061/dryad.15rg7.
