# Supplementary material for: The cost of prospecting for dispersal opportunities in a social bird
Source: Biol Lett. 2016 Jun;12(6):20160316. doi: 10.1098/rsbl.2016.0316 (PMC4938056; doi:10.1098/rsbl.2016.0316)
Supplement: Supplementary methods and results [file rsbl20160316supp1.docx]

**Supplementary material**

**Prospectors: definition and characteristics**

We defined prospectors as individuals captured ≥3 territories from their resident territory to avoid including individuals from adjacent territories attracted to nearby song playback sometimes used to capture birds. In 26 of 27 cases, individuals returned to their resident territory after these prospecting trips; the remaining individual was only caught at the end of the season and not seen again the next season. Although we census territories at least once a week during each season, we may not observe all returned individuals during each visit, so we could not determine the exact duration of prospecting trips. However, of the 26 returned individuals, 18 (69%) were seen in their resident territory in our first (n = 11) or second (n = 7) weekly visit to the resident territory after the prospecting event. The remaining eight individuals were observed inbetween the 3^rd^ and 7^th^ visit. Although the latter may imply that prospecting trips were relatively long for these individuals, they may well have been present but not observed during our visits (e.g. because the primary purpose of the visits after the onset of the breeding season (when a detailed census of all birds is conducted) was to determine nesting behaviour and not the presence of each individual). The duration between catching a prospector and observing it back in its resident territory was on average 13 days (*n* = 17, range = 1-29 days) if they were observed in the same season, and 123 days (*n* = 9, range = 65-168 days) if they were observed back in the next fieldwork period (these individuals were usually caught towards the end of the season). Note that the both estimations are (probably severe) overestimations that rather reflect our census effort than the actual duration of the prospecting trips.

In all cases, both breeders were present in the territory that the prospectors intruded upon, hence ruling out the possibility that these birds were intruding in an attempt to fill a breeding vacancy in that territory.

**Catching protocol and the potential effect of song playback**

Throughout each season, as many birds as possible are caught using mistnets. We aim to catch in all territories on the island, but in the beginning and end of each season we specifically target territories with unringed individuals. Birds are either caught passively or by using song playback (a maximum of three times 15 minutes per territory per day each separated by at least 15 minutes) to attract them to the nets. We did not target prospectors specifically, as we had no prior knowledge or expectation of their presence in a given territory. The mistnets (1-4 per territory, between 6-12m long and 2.5m high) are placed along dense vegetation, in areas sheltered from the wind where possible, and are usually repositioned after a few hours if unsuccessful. The nets are checked for caught birds at least once per 15 minutes. Catching sessions usually last one morning (8:00-12:30) or afternoon (14:00-18:00) per territory. We did not record catching effort, but per season, we, on average (± SE), undertook 47 ± 3.6 days of catching effort (range = 27–72), in 64 ± 2.4 territories (corresponding to catches in 55% ± 2.6 of all available territories (between 109 and 124 territories) with 0.9 ± 0.1 catching days per territory). In most years less intensive fieldwork is also undertaken during the shorter (average ± SE: 38 ± 3 days) and less extensive minor breeding season (December-February) to maintain coverage of the breeding population, but because the data regarding status, residence and helping behaviour at much less accurate/complete during this period we included only main breeding season (June-September) data in this analysis.

In 134 of 254 (53%) cases we caught individuals while song playback was being used. Prospectors were not caught more often with playback (14 of 27 with playback; 52%) than resident birds (120 of 225; 53%; *χ*^2^ < 0.001, *p* = 1.000). Whether or not we used playback did not predict the body mass of individuals (see main text for model details; either alone (*β* = -0.031 ± 0.092; *z* = -0.343, *p* = 0.732) or when included in interaction with whether individuals prospected or not (*β* = -0.193 ± 0.240, *z* = -0.803, *p* = 0.424), suggesting that individuals in better or poorer condition were not more likely to be attracted to the playback. In addition, whether we used song playback did not predict whether individuals were caught together with another individual (see main text for model; *β* = -0.356 ± 0.476; *z* = -0.747, *p* = 0.455). This suggests that the data we present are not biased due to the use of playback. However, we cannot fully exclude that our sampling of prospecting birds may be biased, because differences in the difficulty to catch targeted resident individuals (which may be associated with systematic differences in behaviour) meant that there would have been unequal catching effort in different territories.

**Additional analyses**

***Cross-sectional approach***

**Table S1**. The effect of prospecting behaviour, sex, tarsus length, and the time of day and month of capture on subordinate Seychelles warblers’ body mass (*n* = 214). Results did not change when only the first catch of each individual was included.

|  |  |  |  |  |
| --- | --- | --- | --- | --- |
|  | *β* | SE | *t* | *p* |
| *Intercept* | 5.700 | 1.672 | 3.409 | < 0.001 |
| Prospecting at time of catch^1^ | -0.401 | 0.151 | -2.663 | 0.008 |
| Sex^2^ | 0.813 | 0.139 | 5.870 | < 0.001 |
| Tarsus length | 0.331 | 0.069 | 4.813 | < 0.001 |
| Time^3^ |  |  |  |  |
| Midday | 0.178 | 0.116 | 1.533 | 0.127 |
| Afternoon | 0.660 | 0.119 | 5.563 | < 0.001 |
| Month^4^ |  |  |  |  |
| July | 0.582 | 0.130 | 4.493 | < 0.001 |
| August | 1.077 | 0.139 | 7.731 | < 0.001 |
| September | 0.793 | 0.167 | 4.733 | <0.001 |

^1^Effects relative to ^1^resident birds, ^2^female, ^3^morning, ^4^June

***Longitudinal approach***

To test the possibility that the lower body mass of prospectors observed (see main text) was because such individuals were in poorer condition prior to prospecting, we assessed the within-individual change of body mass of individuals that were caught both in their resident territory and while prospecting. We included individuals with ≤100 days between catches (mean (±SE) = 59 days ±6.7; range: 11-100). In total, 14 individuals (15 catches) were caught in their resident territory and subsequently while prospecting (*n* = 11) or vice versa (n = 4). One individual was caught prospecting once before and once after it was caught in its resident territory but we only included the first comparison for this individual (including only the second comparison instead did not change the results). The within-individual difference in body mass of subordinates caught in their resident territory and while prospecting was assessed using a paired t-test.

In 13 of 14 cases, an individuals’ body mass was lower when caught during prospecting than when caught in their resident territory (binomial sign test: *p* = 0.003); on average (±SE) body mass was 5.27 ± 1.33% (range: -5.3–11.8%) lower during prospecting (paired t-test: *t*_13_ = 3.887, *p* = 0.002; Figure S1). Compared to the cross-sectional analysis we had slightly relaxed the criteria for inclusion to improve sample sizes by including individuals caught in the minor breeding season and individuals between 3 and 5 months old. However, results did not change when excluding the five individuals that were caught once during the minor breeding season (December-February; *t*_8_ = 2.701, *p* = 0.027, average body mass loss = 4.8%), or the four individuals that were between 3 and 5 months old at the time of one or both catches (*t*_9_ = 2.926, *p* = 0.017, average body mass loss = 5.3%).

**Figure S1.** A pairwise comparison of the body mass of 14 subordinate Seychelles warblers caught in their resident territory and while prospecting. On average, body mass was 5.27 ± 1.33% (range: -5.3–11.8%) lower during prospecting (paired t-test: *t*_13_ = 3.887, *p* = 0.002).

***Intraspecific chases***

As individuals are often caught with another individual during intra specific chases (see main text), we assessed whether prospecting and resident individuals (explanatory variable) had a different likelihood of being caught with a resident individual (binomial dependent variable) using a generalized linear mixed model. An analysis with only each individual’s first catch and ‘year’ as random variable yielded a non-significant result (*z* = 1.654, *p* = 0.098). Although this effect was not significant, this test has low power due to a low samples size: the effect size in this subset analysis (23% of 20 prospecting and 8% of 194 residents individuals caught with a resident conspecific; *β* = 1.232 ± 0.745) was similar as for the analysis including all catches (*β* = 1.460 ± 0.539; see main text).
